# Hedinger Syndrome—Lessons Learnt: A Single-Center Experience

**DOI:** 10.3390/jcdd10100413

**Published:** 2023-10-01

**Authors:** Mohamed El Gabry, Sven Arends, Sharaf-Eldin Shehada, Harald Lahner, Markus Kamler, Daniel Wendt, Konstantina Spetsotaki

**Affiliations:** 1 Department of Thoracic and Cardiovascular Surgery, West-German Heart and Vascular Centre, 45147 Essen, Germany; mohamed.elgabry@uk-essen.de (M.E.G.); markus.kamler@uk-essen.de (M.K.); daniel.wendt@cytosorbents.com (D.W.); 2 Department for Anesthesiology and Intensive Care Medicine, University of Duisburg-Essen, 45147 Essen, Germany; sven.arends@uk-essen.de; 3 Department of Endocrinology and Metabolism, University Hospitals Duisburg-Essen, 45147 Essen, Germany; harald.lahner@uk-essen.de; 4 CytoSorbents Europe GmbH, 12587 Berlin, Germany

**Keywords:** Hedinger syndrome, carcinoid heart disease (CD), neuroendocrine tumors (NET), octreotide, tricuspid valve, hemoperfusion

## Abstract

Background: Hedinger syndrome (HS) or carcinoid heart disease (CD) is a rare and challenging manifestation of malignant neuroendocrine tumours (NETs) involving the heart. We aimed to report our experience with surgical strategies and midterm results in HS patients. Methods: Eleven patients (58 ± 11 (range 41 to 79 years); 5 females) with HS who underwent cardiac surgery in our department between 07/2005 and 05/2023 were analysed. Results: All patients showed a New York Heart Association (NYHA) class III–IV and in all the tricuspid valve (TV) was involved. Four patients received a TV replacement, and three TV reconstruction. Recently, to preserve the geometry and function of the compromised right ventricle (RV), we have applied the TV “bio-prosthesis in native-valve” implantation technique with the preservation of the valve apparatus (tricuspid valve implantation: TVI) in four cases. Concomitant procedures included pulmonary valve replacement in four, pulmonary implantation in one, and aortic valve replacement in three cases. To treat RV failure, we adapted a combined TandemHeart^®^-CytoSorb^®^ haemoperfusion strategy in Patient #10 and venoarterial extracorporeal membrane oxygenation (V-A ECMO) support avoidance, after experiencing an ECMO-induced carcinoid-storm-related death in Patient #8. Mortality at 30 days was 18% (2/11). The median follow up was 2 ± 2.1 years (range 1 month to 6 years) with an overall mortality during the follow-up period of 72.7% (8/11). Conclusions: HS surgery, despite being a high-risk procedure, can efficiently prolong survival, and represents a safe and feasible procedure. However, patient selection seems to be crucial. Further follow up and larger cohorts are needed.

## 1. Introduction

Neuroendocrine tumours (NETs) are well-differentiated, rare malignancies, consisting of cells with a neuroendocrine phenotype. Their incidence is steadily increasing at about 6.98/100,000 per year [1,2,3]. Primary sites of NETs can differ, with the most common being the gastrointestinal tract (67.5%), frequently within the small intestine, followed by respiratory (25%), colorectal, the pancreas, and others [1,2,4]. Gastroenteropancreatic NETs (GEP-NETs) consist of gastrointestinal tract carcinoid tumours and pancreatic NETs (pNETs) [5]. NETs are characterized by their metastatic and progressive nature and the wide heterogeneity of inducing symptoms [4]. When metastasized, NETs give rise to the typical carcinoid syndrome, represented by flushing, diarrhoea, and right-sided valvular heart disease (VHD) [5].

Hedinger syndrome (HS) or carcinoid heart disease (CD) is a rare cardiac manifestation of advanced NETs, occurring in about 20–25% of such patients [6,7]. It is related to a broad spectrum of manifestations that include acquired VHD with right-sided, and in rare cases, left-sided involvement, pericardial effusion, and metastases [4,8]. CD is a significant prognosis indicator. Only 31% of patients with CD reach an overall survival of 3 years [7,9]. The underlying pathophysiological cascade is multifactorial and complex, and involves serotonin metabolism and a variety of vasoactive substances released by the hormonally functioning NET itself [6]. Evidence implicates long-term exposure and interaction of the endocardium to high levels of these peptide hormones, mainly serotonin (5-hydroxytryptamine) as one of the key triggers [10]. This results in fibrotic endocardial thickening and the retraction of the valvular and sub-valvular apparatus or even the great vessels. CD involves mostly right-sided heart and valve alterations, causing single or multiple valve lesions, with mixed-stenotic and regurgitative pathology, mostly tricuspid insufficiency and/ or pulmonary stenosis, or even left-sided valve lesions [1,6].

Left-side heart involvement can occur in the presence of septal defects, in the presence of functional lung NETs, and with high levels of vasoactive substances in the systemic circulation that overwhelm the hepatic and pulmonary deactivation capacity [7,11]. Consequently, this myocardial and valve degeneration can be detected in half of the patients, can lead to heart failure, and worsen the already poor prognosis [8]. Corrective cardiac surgery guided by a multidisciplinary approach in selected institutions has gained ground and is effective for symptom relief and survival improvement in highly selected individuals [7,12,13,14]. Although perioperative risk remains higher than that of other pathologies, without an operation, only 15% of symptomatic CD patients survive for 2 years [7,15].

The treatment of CD is challenging as patients frequently present with impaired right heart function, but also with an extensive release of various pro-inflammatory cytokines, resulting in a cytokine storm. The aim of our study was to report our experience with 11 patients with CD after applying a multidisciplinary approach and cardiac intervention, and to provide real-life clinical insights into our strategies and outcomes as our experience with this high-risk patients is expanding.

## 2. Materials and Methods

### 2.1. Data Source and Study Cohort

The present study was a retrospective non-randomized, single-centre analysis. All HS patients that received cardiac surgery at the Department of Thoracic and Cardiovascular Surgery of the Westgerman Heart and Vascular Center in Essen, Germany, between March 2005 and May 2023 were included. Patients’ data, including demographics, clinical findings and results, perioperative assessment, postoperative course, and imaging, were retrospectively extracted from our institutional database. Moreover, survival, cause of death, and functional status were analysed by active follow up. Ethical approval was waived by the local Ethics Committee of the University Duisburg-Essen in view of the retrospective nature of this study and because all procedures being performed were part of routine care.

### 2.2. Perioperative Strategies

We applied an individualized and a multidisciplinary team approach consisting of oncologists, cardiac surgeons, endocrinologists, cardiologists, and anaesthesiologists for each case. Once accepted for cardiac surgery, a standardized medical and surgical protocol was defined for each patient. All cases received an octreotide (Novartis, Basel, Switzerland) infusion of 100 mcg/h 12 h before surgery, which was continued for the first 48 h after surgery. This was followed by subcutaneous octreotide 200 mcg three times a day for the next 14 days. Thereafter, patients were maintained on their long-acting somatostatin analogue. In addition to the octreotide infusion, bolus infusions of steroids and antihistamines were also given to treat the pharmacological symptoms of a carcinoid crisis (tachycardia, bronchospasm, flushing, and labile blood pressure). In the case of pulmonary hypertension, combined therapy with inhaled nitric oxide and iloprost was administrated intraoperatively after weaning from cardiopulmonary bypass (CPB), and until ventilation weaning.

### 2.3. Surgical Methods

In patients undergoing combined procedures, we preferred the median sternotomy approach and standard CPB technique, with ascending aortic and bicaval venous cannulation, mild hypothermia, and cold crystalloid cardioplegic arrest. In cases with isolated tricuspid valve lesion (TVL), surgery was performed under double-lumen endotracheal intubation and femoral cannulation for CPB establishment with a right-sided anterolateral endoscopic approach through the 4th intercostal space. Histological samples of the pericardium and endocardium were obtained in all cases.

### 2.4. Echocardiographic Analysis

**Figure 1:**
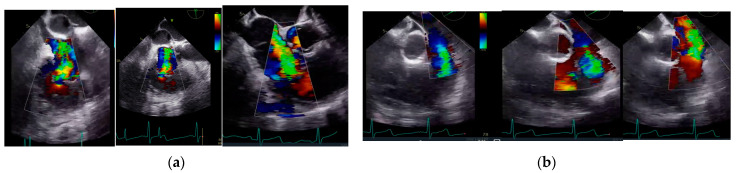
TEE findings prior to surgery, revealing (**a**) severe tricuspid and (**b**) severe pulmonary lesions.

All patients received standardized transoesophageal echocardiography (TEE) before skin incision, immediately after CPB weaning, prior ICU, and during their ICU stay, when indicated. Each TEE was performed with multiplane sector ultrasound transducers (GE 6Tc-RS, 3.0–8.0. MHz or GE 6VT-D, 3.0–8.0. MHz probes) and GE Vivid S6, GE Vivid S70 ultrasound machines (GE Healthcare GmbH, Solingen, Germany) by qualified expert sonographers. Preoperative TEE images of severe affected TV and PV are shown in Figure 1.

## 3. Results

### 3.1. Baseline, Procedural, and Clinical Characteristics

Eleven patients with Hedinger syndrome were included. The mean age was 58 ± 11 years (range 41 to 79 years) and six were male (54.5%). All patients had a New York Heart Association (NYHA) functional class III–IV, with dyspnoea being the most frequent symptom. Nine patients showed moderate to severe right ventricle (RV) dysfunction, and six had decompensated right heart failure with anasarca. The mean EuroSCORE-II was 11.8 ± 10.8%. All patients had severe tricuspid valve lesions. The pulmonary valve (PV) needed intervention in five patients. A left-sided valvular heart disease was present in three patients (27.8%), in two of them caused by a patent foramen ovale (PFO) and in one due to pulmonary metastasis. Detailed baseline characteristics and findings are displayed in Table 1 and Table 2.

### 3.2. Surgery

Seven patients received a conventional median sternotomy and bicaval CPB cannulation, and four patients had an endoscopic approach. Mean CBP and cross-clamp times were 137 ± 45min, and 99 ± 24min., respectively. One patient required re-exploration for bleeding 4 h postoperatively.

### 3.3. Tricuspid Surgical Techniques through the Years

**Figure 2:**
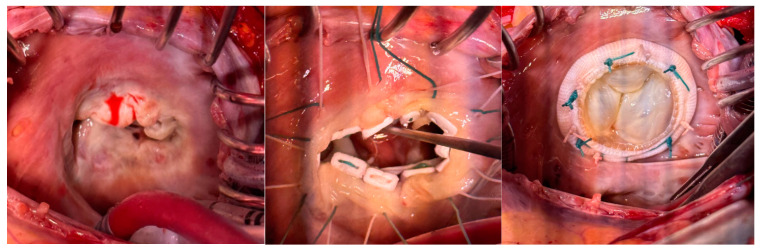
Intraoperative images showing the tricuspid valve implantation (TVI) technique.

In our initial cases, TV replacement was performed after the resection of the native TV. Three patients received a TV repair. Two of these TV repair cases were performed via an endoscopic approach on normothermic beating heart, without any aortic touch or cross clamping. During the study period, we adapted our surgical strategy to the “prosthetic in native” valve-in-valve implantation technique (TVI). This is reflected by splitting the leaflets of the TV as a first step without resection of the anatomical components of the native TV apparatus. This technique allows proper sizing/fitting and preserves the geometry and function of the already compromised right ventricle, by respecting the functional entity of the TV apparatus. Moreover, by laterally folding, buttressing and incorporating the leaflets of the native TV in the mattress suture line, we properly respect the tricuspid effective orifice area and ensure normal prosthetic valve function. An example of a completely affected TV and TVI technique is shown in Figure 2.

### 3.4. Additional Surgical Strategies

**Figure 3:**
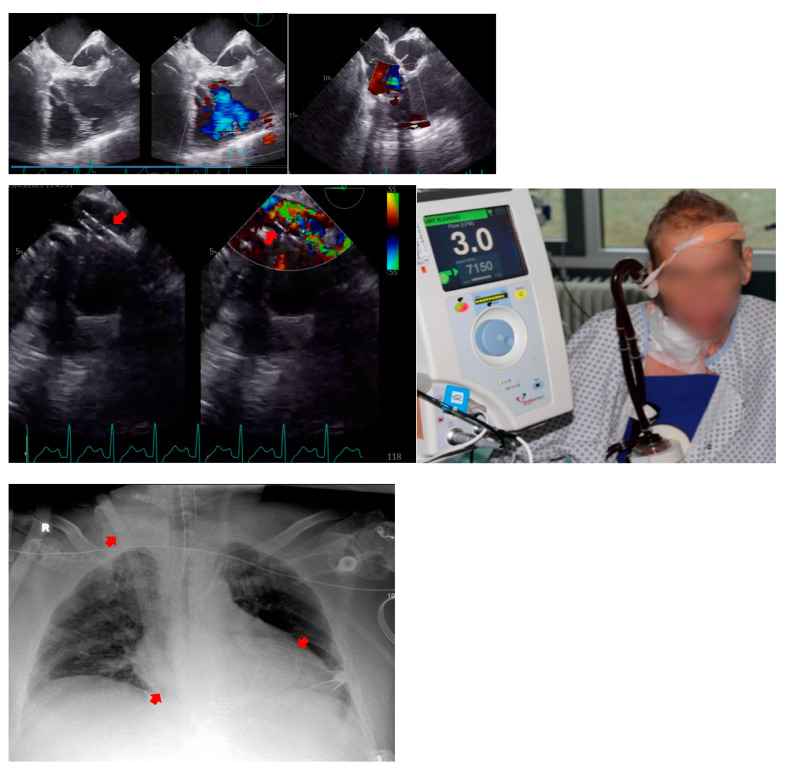
TEE, CXR findings, image of tandem heart of Patient #10 after TVI and TandemHeart ProtekDuo Implantation. Red arrows showing the course of the ProtekDuo (LivaNova, London, UK) double-lumen cannula introduced via the right internal jugular vein.

**Figure 4:**
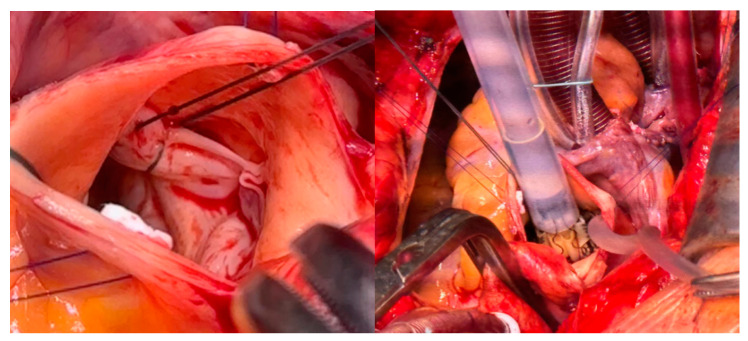
Intraoperative images, showing the Perceval aortic valve replacement (su-AVR) by folding over the leaflets to buttress the aortic valve annulus.

Eight patients (72.7%) received concomitant procedures. The PV was concomitantly operated on in five patients. Two patients had additional RV outflow tract reconstruction (RVOTR) and bovine pericardial patch (BPP) enlargement of the pulmonary artery, and in two patients a pulmonary trunk reconstruction (PTR) was applied. Detailed data are given in Table 2. Patient #8 received an endoscopic TVI with the use of a 31 mm Hancock II prosthesis (Medtronic Inc., Minneapolis, MN, USA) into the preserved insufficient native TV by respecting the sub-valvular apparatus. However, the patient needed venoarterial extracorporeal membrane oxygenation (V-A ECMO) support on the first postoperative day due to severe haemodynamic instability and died 3 days later in carcinoid crisis with fulminant neuroendocrine enzyme and a cytokine storm. Patient #10 received a conventional biological TVI with a 31 mm Hancock II prosthesis, a PFO closure, a RVOTR with BPP and a PVI of a 27 mm Medtronic Freestyle prosthesis. After a reperfusion time of 33 min, a persistent right ventricle (RV) failure occurred. Therefore, a mechanical right ventricular circulatory assist device using a 29 Fr. Protek Duo^®^ cannula inserted into the right internal jugular vein was connected with the para-corporeal TandemHeart^®^ pump (LivaNova, London, UK). The setup is displayed in Figure 3. Patient #11 received a 29 mm Hancock TVI on the preserved native tricuspid valve, PFO closure, and sutureless aortic valve replacement (su-AVR) with an M Perceval Prosthesis, fixated with three sutures, through application of the Cor knot sutureless device (Figure 4).

### 3.5. Clinical Outcomes and Follow Up

One patient died on postoperative day (POD) 1 due to cardiogenic shock. Patient #8 died on POD 4 of a carcinoid crisis which was provoked by the V-A ECMO circulation. Overall 30-day survival was 82% (9/11). The survival data of patients surviving the early postoperative period (>30 PODs) were recorded with a mean follow up of 2 ± 2.1 years (range; 1.5 months to 6 years). Those who survived the early postoperative period showed 1-year survival of 62.5%, 3-year survival of 37.5%, 4-year survival of 37.5%, and 5-year survival of 25%. The causes of death were as follows: five patients died from the primary malignancy (45.5%); two died from cardiogenic shock; and two from another non-cardiac, non-malignancy cause. Thus, the most common cause of death in this cohort was the carcinoid disease itself.

Two patients (18%) showed severe recurrent tricuspid stenosis after repair. We report no re-operations and no cardiac deaths after TVI. Furthermore, in our TVI series, we report no heart-valve complication by means of adhesion, regurgitation/stenosis, or thrombosis of the preserved valvular apparatus to the bio-prosthesis. All our patients showed clear functional improvement with an NYHA I–II downgrade and improvement of life quality after TVI.

Additionally, we reported no postoperative valve thrombosis complications in our cohort. The postoperative anticoagulation strategies, in this cohort, were not only based on guidelines and consensus for anticoagulation in patients with carcinoid heart disease, but also further individualized for each case, taking into consideration operative and postoperative surgical aspects (surgical technique used, combined valve procedures, type of prosthesis used in the case of replacements, anatomical side involved, postoperative surgical bleeding, thrombosis, other planned surgeries), risk of acute thrombosis, and hypercoagulability aspects due to carcinoid disease and coagulopathies triggered by high levels of serotonin, hypercoagulable status before surgery, platelet aggregation, liver metastasis, liver pathologies, and other comorbidities. By taking all the above into consideration, and in the absence of any contraindications, the routine anticoagulation included Vitamin K antagonists for the first 3 postoperative months and afterwards 100 mg aspirin as a lifelong monotherapy unless contraindicated. Detailed postoperative findings and results are seen in Table 3. 

## 4. Discussion

In the present study, we report our experience with the rare and demanding cardio-surgical treatment of Hedinger patients, our institutional concepts, and their potential benefits once combined with careful patient selection. The complicated entity of CD is one of poor prognosis, and cardiac surgery in such patients remains a high risk. The actual operative risk cannot be estimated by applying conventional risk scores used in cardiac surgery, as these do not take into account the malignancy and cardiac disease risk aspects. Small intestinal NETs can release various vasoactive substances, such as serotonin (5-hydroxytryptamine; 5-HT), tachykinins, prostaglandins, histamine, kallikrein, and others. When released into the portal circulation, these can be inactivated through the liver [6,16,17]. However, when a serotonin-producing NET metastasizes, direct access of the above substances into the systemic circulation can induce carcinoid syndrome [6,16,17], and the development of the paraneoplastic CD [18,19]. CD is characterized by fibrosis of the endocardium, valve apparatus, cardiac chambers, and even the intima of great vessels, such as the pulmonary arteries and the aorta [20]. We have highlighted and confirmed the importance of a multidisciplinary team approach, careful preoperative evaluation, and the definition and individualization of the most appropriate treatment algorithm early before surgery [14,21]. Thus, cardiac surgery in such high-risk patients should always be reserved for highly specialized centres that can offer such an experienced team approach, consisting of cardiologists, tumour specialists, anaesthesiologists, and endocrinologists. Moreover, we underline the need and significance of combined or even modified surgical techniques that also consider the impaired right heart dysfunction.

In our cohort, the pulmonary valve was involved in six cases. Of these, in three patients, the pulmonary artery was fibrotic and stenotic and needed to be enlarged with pericardial patch plastic. Left-sided heart valves were also involved in three patients.

Although reports on the surgery for CD are increasing, there is still scant data on the surgical approach and postoperative results. Our study covers a time interval of 18 years, and shows a wide postoperative survival range from one day (during our early experience) to, at present, six years. We showed an overall 30-day mortality rate of 18% (2/11). However, in recent years, we have reported zero perioperative and early postoperative mortality. Overall mortality during the study interval was 72.7% (8/11). Our results are comparable to the groups of Collony, Yong, Honan, and others [7,13,22,23,24]. The overall postoperative survival at 1, 3, and 5 years was reported as 69%, 48%, and 34%, based on a Mayo Clinic series evaluating 240 patients [7,24].

In carefully selected CD patients, surgical intervention is considered for the palliation of heart-failure symptoms, and for improvement of prognosis and quality of life [25,26,27]. The surgical results of CD are improving, with lower operative mortality related to prompt referral and expanding experience in the field [28]. However, long-term survival remains poor due to the progression of CD, rather than the cardiac surgery per se, in these patients [25,29]. Most major complications are RV dysfunction, uncontrolled or refractory CD, carcinoid crisis, and coagulopathies [30].

Another serious postoperative complication might be the recurrence of valvular heart disease or structural valve deterioration [23]. Two of our patients showed significant postoperative valve or prosthesis degeneration and required re-intervention during the late postoperative period. During the present follow up, the recurrence of tricuspid valve disease was naturally more likely in repaired than in replaced patients. This is consistent with other studies and the European Neuroendocrine Tumor Society’s (ENETS) 2022 guidance [7,23]. Consequently, we changed our strategy and adapted as the treatment of choice the bio-prosthesis valve-in native valve implantation (TVI) technique with the preservation of the valvular apparatus for the pulmonary and tricuspid valve lesions. Moreover, since our first discouraging experience with V-A ECMO support in Patient #8, which triggered a fulminant carcinoid/cytokine storm, it is now our institutional policy to strictly avoid V-A ECMO support [31]. As an alternative, we now use the temporary percutaneous right-sided assist pump TandemHeart^®^ via a Protek Duo^®^(LivaNova, London, UK) double-lumen cannula introduced via the right internal jugular vein to establish right ventricular mechanical circulatory support. By doing so, we preserve the natural and protective barrier of pulmonary metabolism, which usually avoids the left-sided spread of carcinoid substances. Moreover, as another adjunctive option, we recently combined the TandemHeart^®^ with extracorporeal haemoadsorption (Cytosorb^®^ Inc., Princeton, NJ, USA) which offers a potent method for cytokine adsorption in carefully selected cases. The paradigm of Patient #10’s management and some findings are presented in Figure 3 below. To the best of our knowledge, this combined treatment is the first paradigm of cardio-surgical management of its kind for a selected critical patient with Hedinger syndrome, and could be potentially used as a valuable therapeutic option, or a rapid treatment to restore right ventricular failure and avoid the catastrophic results of carcinoid storm triggered by other circulatory devices such as ECMO. However, it is crucial to consider the optimal patient selection, optimal timing of implantation to avoid irreversible organ damage, the optimal support device to ensure the right mechanism of action, and the availability and expertise of the centre.

## 5. Conclusions

The multidisciplinary team approach combined with patient-tailored surgical strategies, such as modified native valve-apparatus-saving techniques, is of utmost importance in CD patients. We observed a prolongation of survival and an improvement of functional status (from NYHA III–IV to NYHA I) and quality of life. Moreover, we report our encouraging results using the right-sided Tandem-Heart^®^ instead of VA-ECMO circulatory support in cases of acute RV failure. In addition to this, adjunctive haemoadsorption during the use of Tandem-Heart^®^ support might be another adjunctive therapy to mitigate the cytokine storm. Although recent developments have reached an improvement of the poor prognosis of this entity, further multi-centre studies with larger cohorts and longer follow-up data are needed to confirm our observations and their potential in clinical practice.

## Figures and Tables

**Table 1:** Preoperative patient characteristics.

	Case 1	Case 2	Case 3	Case 4	Case 5	Case 6	Case 7	Case 8	Case 9	Case 10	Case 11
Demographics											
Age, years	41	60	49	65	54	54	79	74	67	45	55
Female gender	Yes	No	Yes	No	No	No	Yes	Yes	Yes	No	No
PTL	SI	SI	SI	SI	SI	SI	SI	SI	SI	SI	SI
Metastasis	Yes	Yes	Yes	Yes	Yes	Yes	Yes	Yes	Yes	Yes	Yes
COPD	No	No	No	Yes	Yes	No	Yes	Yes	No	No	No
Hypertension	Yes	Yes	Yes	Yes	Yes	Yes	Yes	Yes	Yes	Yes	No
AF	No	Yes	No	No	No	No	No	No	No	No	No
DM Type II	Yes	No	Yes	No	No	No	Yes	Yes	No	Yes	No
HLP	No	Yes	No	No	No	Yes	Yes	Yes	No	No	No
RD (>200 µmol/L)	No	No	Yes	Yes	Yes	Yes	Yes	Yes	No	No	No
EuroSCOREII (%)	3.7	4.3	8.7	36.1	6.1	2.4	29.8	18.1	6.4	8.6	5.4
NYHA	III	IV	III	IV	III	IV	III	IV	IV	IV	IV
PCS	No	No	No	Yes	No	No	No	No	No	No	No
PAH	No	No	Yes	Yes	Yes	Yes	Yes	Yes	No	No	No
Preoperative echocardiographic findings											
LV-EF (%)	55	57	45	40	45	50	55	50	50	50	55
TVL	Yes	Yes	Yes	Yes	Yes	Yes	Yes	Yes	Yes	Yes	Yes
PVL	Yes	Yes	Yes	No	No	Yes	No	No	Yes	Yes	No
MVL	No	No	No	Yes	No	No	No	No	No	No	No
AVL	No	No	No	Yes	Yes	No	No	No	No	No	Yes
PFO	No	Yes	No	No	No	No	No	No	No	Yes	Yes
IRVF	Moderate	Moderate	Moderate	Moderate	Moderate	Severe	Moderate	Severe	No	Severe	No

AF atrial fibrillation, COPD chronic obstructive pulmonary disease; DM diabetes mellitus; NYHA New York Heart Association; LV-EF left ventricular ejection fraction; TVL tricuspid valve lesion; PVL, pulmonary valve lesion; MVL mitral valve lesion; AVL aortic valve lesion; HLP hyperlipidaemia; IRVF impaired right ventricular function; PAH pulmonary arterial hypertension; PFO patent foramen ovale; PCS previous cardiac surgery; PTL primary tumour location; RD renal disease.

**Table 2:** Intra- and postoperative procedural characteristics.

	Case 1	Case 2	Case 3	Case 4	Case 5	Case 6	Case 7	Case 8	Case 9	Case 10	Case 11
Valvular Lesions Before Surgery											
TV	Combined	Combined	Combined	Severe Regurgitation	Combined	Combined	Severe Regurgitation	Severe Regurgitation	Severe Regurgitation	Combined	Combined
PV	Combined	Combined	Severe Regurgitation	No	No	Combined	No	No	Severe Regurgitation	No	No
MV	No	No	No	Severe Regurgitation	No	No	No	No	No	No	No
AV	No	No	No	Severe regurgitation	Severe regurgitation	No	No	No	No	No	Severe Regurgitation
Surgical Strategy											
TV	Replacement	Replacement	Replacement	Repair	Repair	Replacement	Repair	Implantation	Implantation	Implantation	Implantation
PV	Replacement	Replacement	Replacement	No	No	Replacement	No	No	Implantation	No	No
MV	No	No	No	Repair	No	No	No	No	No	No	No
AV	No	No	No	suAVR	suAVR	No	No	No	No	No	suAVR
RVOTR	No	No	No	No	No	No	No	No	Yes	Yes	No
PTR	No	No	Yes	No	No	Yes	No	No	No	Yes	No
PFO closure	No	Yes	No	Yes	No	No	No	No		Yes	Yes
Assist Device needed	No	No	No	No	No	No	No	Yes; v.a.ECMO	No	Yes; TandemHeart	No
XCL, min	95	78	79	74	90	124	0	0	95	107	84
CPB time, min	131	120	146	101	123	256	74	87	118	151	90

CPB cardiopulmonary bypass; PTR pulmonary trunk reconstruction; RVOTR right ventricle outflow tract; V-A ECMO venoarterial extracorporeal mechanical oxygenation; XCL aortic cross-clamp time.

**Table 3:** Postoperative Complications and Follow up.

Variable	Case 1	Case 2	Case 3	Case 4	Case 5	Case 6	Case 7	Case 8	Case 9	Case 10	Case 11
AV Block III	Yes	No	No	No	No	No	No	No	No	No	No
PPI	Yes	No	No	No	No	No	No	No	No	No	No
AKNI (Dialysis)	No	Yes	Yes	Yes	No	Yes	No	Yes	No	Yes	No
Delir	No	No	No	Yes	No	No	No	No	No	No	No
Redo (Major Bleeding)	No	Yes	No	No	No	No	No	No	No	Yes	No
Pneumonia	No	No	No	No	No	No	No	No	No	Yes	No
Sepsis	No	Yes	No	No	No	No	No	No	No	Yes	No
30-day Survival	Yes	No	Yes	Yes	Yes	Yes	Yes	No	Yes	Yes	Yes
Re-Do (Heart Valve Lesion)	No	No	No	No	Yes, Recurrent TV stenosis	No	Yes, Recurrent TV stenosis	No	No	No	No
Status at Last Follow up	Dead	Dead	Dead	Alive, NYHA I–II	Dead	Dead	Dead	Dead	Alive, NYHA I–II	Dead	Alive
Survival *	15 Months	1 Day	18 Months	72 Months, Alive	60 Months	4 Months	6 Months	4 Days	50 Months, Alive	1.5 Month	1.5 Month, Alive
Cause of death	Carcinoid Disease	Cardiogenic Shock	Accident		Carcinoid Disease	Carcinoid Disease	Accident	Carcinoid Disease		Carcinoid Crisis	

AKI acute kidney injury; CPB cardiopulmonary bypass; PPI permanent pacemaker implantation; TV tricuspid valve, * Survival time is defined as time from surgery to last follow up (May 2023)/death.

## Data Availability

Data available on request due to privacy restrictions. The data presented in this study are available on request from the corresponding author. The data are not publicly available due to privacy concerns.

## Abbreviations

AF	atrial fibrillation
AKI	acute kidney injury
AVL	aortic valve lesion
CD	carcinoid disease
CBP	cardiopulmonary bypass
COPD	chronic obstructive pulmonary disease
CS	carcinoid syndrome
DM	diabetes mellitus
ENETS	European Neuroendocrine Tumor Society
GEP-NETs	gastroenteropancreatic NETs
HLP	hyperlipidaemia
IRVF	impaired right ventricular function
LV-EF	left ventricular ejection fraction
MVL	mitral valve lesion
NETs	neuroendocrine tumours
NYHA	New York Heart Association
PAH	pulmonary arterial hypertension
PCS	previous cardiac surgery
pNETs	pancreatic NETs
PFO	patent foramen ovale
PPI	permanent pacemaker implantation
PTR	pulmonary trunk reconstruction
PTL	primary tumour location
PV	pulmonary valve
PVL	pulmonary valve lesion
RD	renal disease
RVOTR	right ventricle outflow tract
TOE	transoesophageal echocardiogram
TVL	tricuspid valve lesion
TVI	tricuspid valve implantation
V-A ECMO	veno-arterial extracorporeal mechanical oxygenation
VHD	valvular heart disease
XCL	aortic cross-clamp time
